# The Selective Mutism Questionnaire: Data from typically developing children and children with selective mutism

**DOI:** 10.1177/1359104520914695

**Published:** 2020-04-13

**Authors:** Beate Oerbeck, Kristin Romvig Overgaard, R. Lindsey Bergman, Are Hugo Pripp, Hanne Kristensen

**Affiliations:** 1 Division of Mental Health and Addiction, Oslo University Hospital, Norway; 2 UCLA Semel Institute for Neuroscience and Human Behavior, USA; 3 Oslo Centre for Biostatistics and Epidemiology, Oslo University Hospital, Norway; 4 Regional Centre for Child and Adolescent Mental Health, Eastern and Southern Norway, Norway

**Keywords:** Selective mutism, Selective Mutism Questionnaire, School Speech Questionnaire, typically developing children

## Abstract

The core symptom of the anxiety disorder selective mutism (SM) is absence of speech in specific situations, such as at school. The most commonly used standardized instruments to assess speaking behavior are the parent-rated Selective Mutism Questionnaire (SMQ) and the teacher-rated School Speech Questionnaire (SSQ), scored from 0 to 3, indicating that speaking behavior never, seldom, often, and always occur. They were developed to assess severity of mutism and potential effects of treatment. However, prospective data on speaking behavior in typically developing children (TDs) are missing in the literature. The main aim of this study was to present data from TDs over time with previously reported data from children treated for SM, as a comparison. Participants were 64 children aged 3–9 years, 32 TDs who were a matched control group to 32 children with SM. At baseline, the mean SMQ and SSQ scores were ⩾2.5 in TDs and 0.5 in children with SM. The TDs did not show significant changes over time, while significantly increased speech was found in children with SM after treatment. Thus, our findings support the use of the SMQ/SSQ to assess baseline SM severity and to evaluate potential treatment effects in future studies.

Selective mutism (SM) is characterized by a consistent lack of speech in specific social situations in which there is an expectation for speaking (e.g., at school), despite speaking freely in other situations (e.g., at home with close family members). The diagnostic criteria specify that the failure to speak is not attributable to a lack of knowledge of, or comfort with, the spoken language required in the social situation. Furthermore, the disturbance is not better explained by a communication disorder (e.g., childhood-onset fluency disorder) and does not occur exclusively during the course of autism spectrum disorder, schizophrenia, or another psychotic disorder. SM is an impairing anxiety disorder with age of onset generally during early preschool years and a point prevalence between 0.03% and 1% (American Psychiatric Association [APA], 2013). Historically, the term was *elective* mutism, suggesting that mutism was a deliberate act. Based on increasing research on the association between SM and anxiety disorders in general, and SM and social anxiety disorder (SAD) in particular, SM was classified as an anxiety disorder in the *Diagnostic and Statistical Manual of Mental Disorders* (DSM-5; APA, 2013). This reflects a shift in the understanding of SM from an act of will to a lack of ability to speak in select situations, considered to be due to anxiety. Another link to anxiety is demonstrated by children with SM having been characterized with a behaviorally inhibited temperament (Gensthaler et al., 2016; Muris et al., 2016), a known risk factor for anxiety disorders (Bufferd et al., 2018).

The Selective Mutism Questionnaire (SMQ; Bergman et al., 2008) and the School Speech Questionnaire (SSQ; Bergman et al., 2002) are the most widely used standardized instruments for assessing SM symptoms. They were designed to provide a quantitative measure of severity, scope, and impairment related to SM. Parents use the SMQ, which includes subscales measuring speech in three different contexts; at school, at home, and in public, while teachers use the SSQ, a measure of speech at school. The SMQ and SSQ severity scoring ranges from 0 (never speaking) to 3 (always speaking). The measures are not diagnostic tools, but were developed to assess treatment effects, and therefore do not yield cut-off scores. However, the author’s initial psychometric study (Bergman et al., 2008) and subsequent research studies have generated “benchmark” SMQ values often used for comparison by authors of empirical investigations. In the Bergman study (Bergman et al., 2008), a baseline score ⩽0.5 was found on the SMQ School subscale for children with SM, with no significant gender differences. In that study, which included children with SM in three age groups (3–5, 6–8, and 9–11 years), children in the oldest age group had lower SMQ scores than the youngest age group. An independent psychometric study supported the SMQ three-factor solution (at school, at home, and in public) while age and gender differences were not found (Letamendi et al., 2008). That study also reported internal consistency (α = .78) for the total SMQ score. Furthermore, support for the convergent validity was demonstrated in a correlation (*r* = .42) between the SMQ and a clinician-rated severity measure of SM from the Anxiety Disorders Interview Schedule (ADIS-IV; Albano & Silverman, 1996). Incremental validity was demonstrated when the SMQ added significant variance in the prediction of SM diagnosis over and above the CBCL Anxious/Depressed Subscale (Achenbach et al., 2003). Specifically, this relationship was driven by the Public (β = .06, *p* = .001) and the School subscales (β = .03, *p* = .006) for preschoolers and the School subscale (β = .06, *p* < .001) for schoolchildren.

Baseline scores below 1 on the SSQ and the SMQ School subscale have largely been supported in data from recent SM treatment studies which include a reasonably large samples of children, in both United States and Europe (Bergman et al., 2013; Catchpole et al., 2019; Cornacchio et al., 2019; Klein et al., 2017; Lang et al., 2016; Oerbeck et al., 2014) (see Table 1). These studies also demonstrate small, but significant changes after treatment (of different lengths) with post-treatment scores ranging from 1 to 3 (generally ⩽2).

**Table 1.** Baseline SMQ and SSQ scores in published selective mutism treatment studies.^a^

Measure	Bergman et al. (2013)^b^ *n* = 27, 48% girls, age 4–8	Oerbeck et al. (2014) *n* = 24, 65% girls, age 3–9	Lang et al. (2016) *n* = 24, 50% girls, age 3–13	Klein et al. (2017) *n* = 33, 62% girls, age 5–12	Catchpole et al. (2019) *n* = 31, 52% girls, age 4–10	Cornacchio et al. (2019) *n* = 29, 76% girls, age 5–9
	*M* (*SD*)	*M* (*SD*)	*M* (*SD*)	*M* (*SD*)	*M* (*SD*)	*M* (*SD*)
SMQ at school	0.38 (0.35)	0.50 (0.40)	0.52 (0.99)	0.53 (0.68)	0.67 (0.56)	Missing
SMQ at home	1.73 (0.66)	1.65 (0.64)	1.63 (1.15)	2.04 (0.46)	1.88 (0.67)	1.90 (0.70)
SMQ in public	0.48 (0.67)	0.33 (0.43)	0.42 (0.83)	0.33 (0.37)	0.26 (0.34)	0.70 (0.60)
SMQ total	0.85 (0.38)	0.86 (0.35)	0.88 (1.15)	0.98 (0.39)	0.96 (0.44)	Missing
SSQ	0.64 (0.54)	0.55 (0.43)	Missing	Missing	0.61 (0.56)	1.10 (0.70)

SD: standard deviation; SMQ: parent-rated Selective Mutism Questionnaire; SSQ: teacher-rated School Speech Questionnaire.

a Included are studies with a reasonably large sample reporting data following the SMQ/SSQ scoring instructions.

b Reported here are baseline data on *n* = 27 (courtesy of Lindsey Bergman, as the Bergman 2013 study report data on *n* = 21).

In the initial psychometric study of the SMQ, a subset of children with SM (*n* = 11) received 28 sessions of behavioral treatment (Bergman et al., 2008). The treatment resulted in significantly increased scores on the SMQ, taken as preliminary evidence for the validity of the measure as sensitive to effects of treatment. More recently, two randomized controlled trials (RCTs) showed no significant change in the amount of speaking, as measured by the SSQ and SMQ in waitlist controls with SM after 3 months, while a significant increase was found in children with SM after 3 months of treatment (Bergman et al., 2013; Oerbeck et al., 2014). In one of these RCTs, significantly increased speech was found at the end of 6 months treatment for SM, and treatment gains were upheld at follow-up 2 year after end of treatment (Oerbeck et al., 2015). These findings were taken as additional support for the sensitivity of the instruments to treatment effects. Scores on the SMQ School subscale for a small group of children (*n* = 18) referred for anxiety disorders other than SM were found to be ⩾2.5 (Bergman et al., 2008). However, that sample was small and did not include a subgroup of children with SAD. Whether SM and SAD represent two distinct conditions is still not settled. Although most descriptive and controlled studies using diagnostic interviews have found high rates of comorbidity between SM and SAD (61–100%; Chavira et al., 2007; Manassis et al., 2007), there have been studies with low rates of SAD in SM (⩽18%; Carbone et al., 2010; Edison et al., 2011; Nowakowski et al., 2011). Children with SAD may also show speech problems, such as longer speech latency, inappropriate tone or low voice volume, and reduced spontaneous speech (Beidel et al., 2019). Whether speaking behavior differentiates the two conditions has not been evaluated until the recent publication of the Frankfurt Scale of Selective Mutism (FSSM; Gensthaler et al., 2018). The parent-rated FSSM is a validated, age-adjusted measure of SM for both research and clinical practice and includes a diagnostic scale (DS) and a severity scale for three age groups (3–18 years). In a study including children and adolescents with SM, SAD, or internalizing disorders, and a control group (*n* = 334), the mean DS sum scores differed significantly between the diagnostic groups, and the receiver operating characteristic analysis gave optimal cutoffs for distinguishing SM from the other groups with the area under the curves of 0.94–1.00 (Gensthaler et al., 2018).

Previous research has found that the SMQ distinguished children with SM from control children, but the *N* was low (16 and 19 controls) (Bar-Haim et al., 2004; Manassis et al., 2007). However, data on the teacher rated SSQ, as well as SMQ data on prospective speaking behavior in typically developing children (TDs), are missing in the literature. Such data could clarify whether TDs have variability in speaking behavior across settings and over time, and whether age and gender differences are present. Clarification on these issues can help establish additional validity for the SMQ and SSQ and increase our confidence in their use for research on the assessment and treatment of SM.

This study expands previous research by investigating speaking behavior, including possible age and gender differences in TDs over time (after 6 and 18 months, the time period most often investigated in SM treatment and follow-up studies), with previously reported data from children treated for SM, as a comparison. Based on the two studies mentioned, we assumed that TDs would show significantly more speech at baseline on the SM questionnaires than children with SM. As the questionnaires primarily measure whether children actually talk and/or answer in certain situations, we further hypothesized no significant changes over time in TDs.

## Methods

### Participants

Participants were 64 children from two groups: one group consisted of TDs who were a matched control group to a group of children treated for SM. Data from the children with SM have previously been reported (see below). The principal investigators met all the families at home. The assessment of the group of TDs took place after having completed the assessment and treatment of the SM group (within 1 year).

Group 1 consisted of 32 children with SM, 3–9 years of age who completed a home- and school-based intervention for SM after referral from outpatient Child and Adolescent Mental Health Clinics (CAMHS) or school psychology services all over Southern Norway. Included were 11 boys and 21 girls, 16 preschool children; age 3–5 years and 16 schoolchildren; age 6–9 years who started treatment by local therapists under guidance from the first and last author at mean age 6.11 years (*SD* = 1.97). This group is comprises 7 children from our pilot study (Oerbeck et al., 2012), 24 children from our RCT study (Oerbeck et al., 2014), and 1 child not included in the RCT who received the same treatment for SM by a therapist in the RCT study during the same time period. None used medication.

All fulfilled the DSM diagnostic criteria for SM by the use of the SM module from the clinician-administered semi-structured ADIS-IV (Albano & Silverman, 1996). The reliability and validity of the ADIS-IV-C/P has been established (Silverman et al., 2001; Wood et al., 2002). In line with the DSM-IV, the ADIS-IV-C/P includes questions regarding both the child’s symptoms and functional impairment, that is, a clinical severity rating (CSR; range = 0–8), and children are assigned diagnoses when the CSR ⩾4). The SM module relates to the speaking behavior of the child in different social situations, and we used the CSR cut point (4). In addition, we specified that the children did not speak to adults in preschool/school, and that mutism also was present in both languages for bilingual children.

Comorbid diagnoses were assessed with the revised version of the Schedule for Affective Disorders and Schizophrenia for School-Aged Children: Present and Lifetime Version (K-SADS-PL; Kaufman et al., 1997). At baseline, all 32 children also fulfilled criteria for social phobia. Additional diagnoses were found in 20/32 children (63%) including separation anxiety disorder (*n* = 10), specific phobia (*n* = 6), generalized anxiety disorder (*n* = 2), obsessive-compulsive disorder (*n* = 2) transient tic disorder (*n* = 3), enuresis nocturna (*n* = 6), and encopresis (*n* = 1). In all, 10 children were bilingual. The educational level of the 64 parents (mothers and fathers) was ⩽12 years (*N* = 30) and >12 years (*N* = 34; 12 years of schooling is equivalent to completing a high school education).

Group 2 consisted of a matched control group of 32 TDs, 3–9 years of age (21 girls, mean age = 6.12 years (*SD* = 1.99)), 16 preschool children, and 16 schoolchildren). They were all non-referred children, consecutively recruited as normally functioning children by teachers, who knew them well. They were matched to each participating child with SM with regard to age, gender, ethnicity (and being bilingual), and parental education. For this group, diagnostic interviews were not done, but mothers completed brief interviews regarding their child’s medical and developmental history, behavioral and psychological functioning, and no significant problems were reported. (Examples of questions from the interview: “Does the child have any medical illness?” “Compared to peers, do you think your child’s development has been advanced, normal or delayed?” (asked separately for motor function, attention and language); “Compared to peers, do you think your child’s behavior and psychological functioning is age-appropriate?”). In all, 10 children were bilingual. The educational level of the 64 parents of the TDs (mothers and fathers) was ⩽12 years (*N* = 28) and >12 years (*N* = 36), not significantly different from the SM group. None used medication.

Written informed consent was provided by the parents. The study was granted approval by the Norwegian Social Science Data Services and the Regional Committees for Medical and Health Research Ethics (South East Norway).

### Measures

For both groups, two SM questionnaires were completed by parents and teachers at three time points: baseline (before treatment for the children with SM), after 6 months (end of treatment for the children with SM), and after 18 months (1 year after end of treatment for the children with SM).

The SMQ (Bergman et al., 2008) was completed by the mothers. The principal investigators sent the SMQ to the parents by mail and received them back by mail. The SMQ includes 32 items scored from 0 to 3, where 0 indicates that speaking behavior never occurs, and 1, 2, and 3 refer to seldom, often, and always speaking, respectively. In total, 17 SMQ items were used to compute the three subscale mean scores, at school (six items), at home (six items), and in public (five items), computed as the mean of the relevant items, and the SMQ total score is the sum of the three subscales. We present the SMQ scores divided by the number of relevant items to express the scores in the same range as each item is rated—from 0 to 3. In this study, we used the approved Norwegian translation, available at https://iacapap.org/content/uploads/F.5-MUTISM-NORWEGIAN-2019.pdf. Internal consistency (α) on the SMQ total score was .76 in the TDs and .77 (both acceptable) in children with SM. When increasing the number of participants by including all (*n* = 64), and thus increasing the variance, α was excellent (.96).

The SSQ (Bergman et al., 2002) was completed by teachers. The principal investigators sent the SSQ to the teachers by mail, and the teachers mailed them back, independent of the parents. The SSQ is based on frequency of speech at school and includes 10 items modified from the SMQ to suit teachers. Six of the SSQ items (identical to the SMQ School subscale) were used to express the score in the same 0–3 range used in each item. As in the SMQ, a score 0 indicates that speaking behavior never occurs, and 1, 2, and 3 refer to seldom, often, and always speaking, respectively. In this study, we used the approved Norwegian translation, available at https://iacapap.org/content/uploads/F.5-MUTISM-NORWEGIAN-2019.pdf. Internal consistency (α) was .80 (acceptable) in the TDs and .66 (questionable) in children with SM. When increasing the number of participants by including all (*n* = 64), and thus increasing the variance, α was excellent (.97).

### Data analysis

Descriptive statistics using means and error bars with 95% confidence intervals (CIs) are presented for the SM questionnaires (SMQ, SSQ) over time at baseline (T1), after 6 months (T2), and 18 months (T3) for the two groups (SM and TDs).

Linear mixed models for repeated measurements using a subject-specific random intercept were applied to investigate potential changes from T1 through to T3 in the two groups and differences between the groups. We also checked for possible age and gender effects by including them and relevant interaction terms between groups, follow-up time, and covariates in the models. Mean differences were estimated from marginal means, and the *p* values were Bonferroni-corrected. The level of significance was defined as *p* < .05.

## Results

Linear mixed models for repeated measurements found that TDs showed significantly more speech, as rated by the SM questionnaires (SMQ, SSQ) at baseline (T1), T2, and T3 compared to children with SM (*p*’s < .001). Over time (T1–T3), there were no significant changes in TDs, while there was a significant change from T1 (baseline) to T2 (after 6 months of treatment) in children treated for SM (*p*’s < .001).

**Figure 1.**
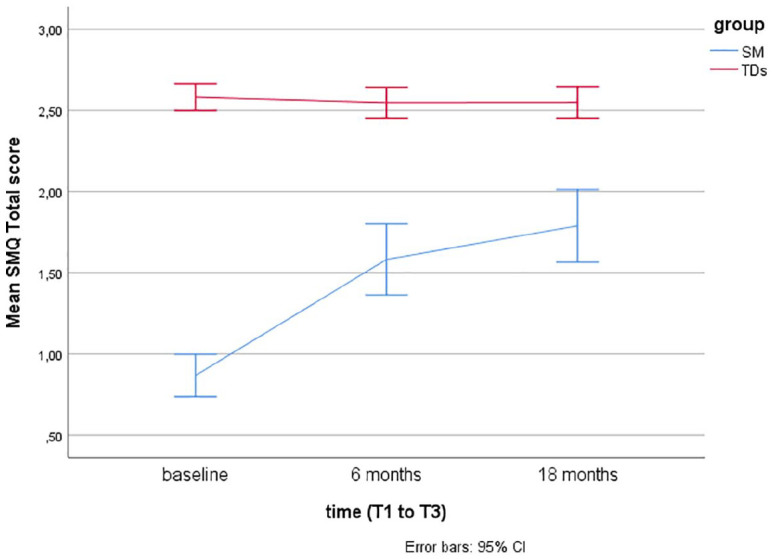
Mean Selective Mutism Questionnaire (SMQ) total scores over time (T1–T3) in typically developing children (TDs) and children treated for selective mutism (SM).

**Figure 2.**
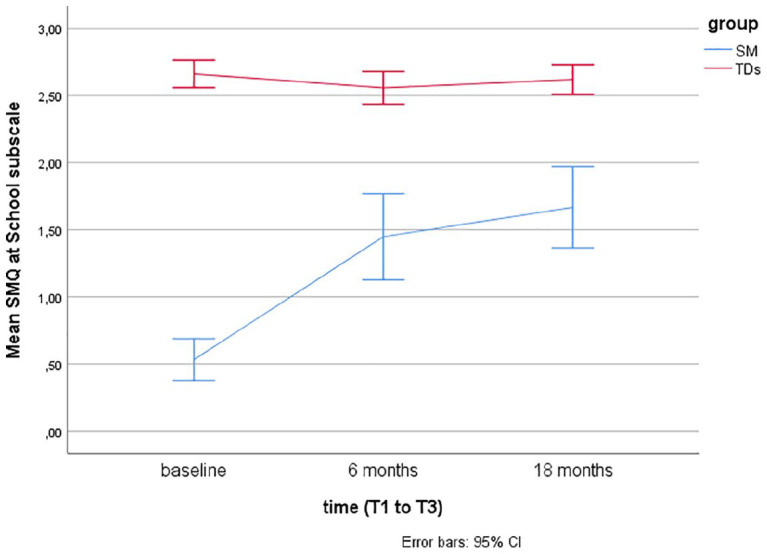
Mean Selective Mutism Questionnaire (SMQ) School subscale scores over time (T1–T3) in typically developing children (TDs) and children treated for selective mutism (SM).

**Figure 3.**
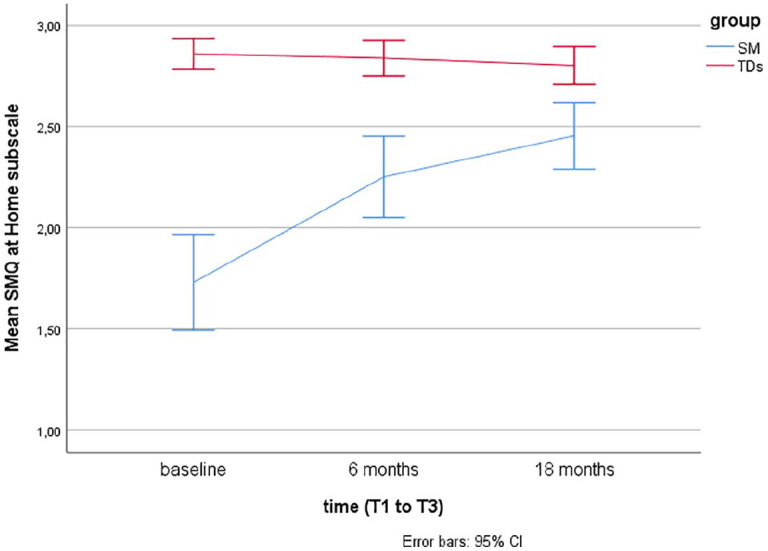
Mean Selective Mutism Questionnaire (SMQ) Home subscale scores over time (T1–T3) in typically developing children (TDs) and children treated for selective mutism (SM).

**Figure 4.**
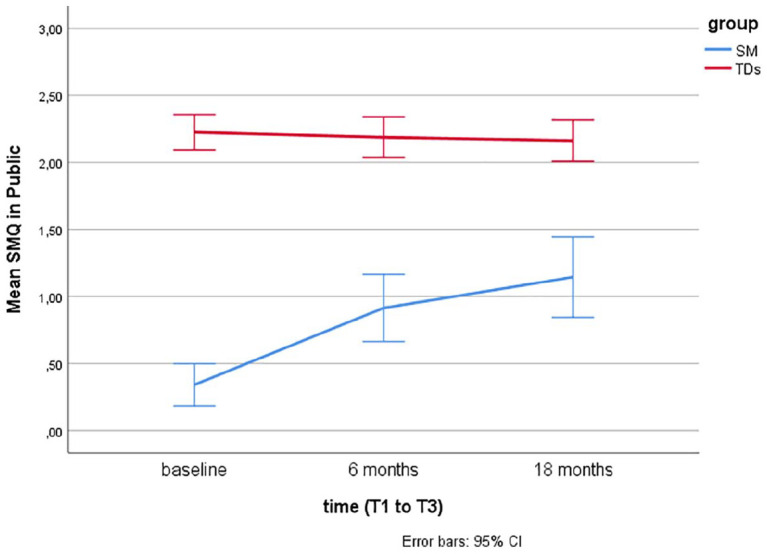
Mean Selective Mutism Questionnaire (SMQ) Public subscale scores over time (T1–T3) in typically developing children (TDs) and children treated for Selective Mutism (SM).

**Figure 5.**
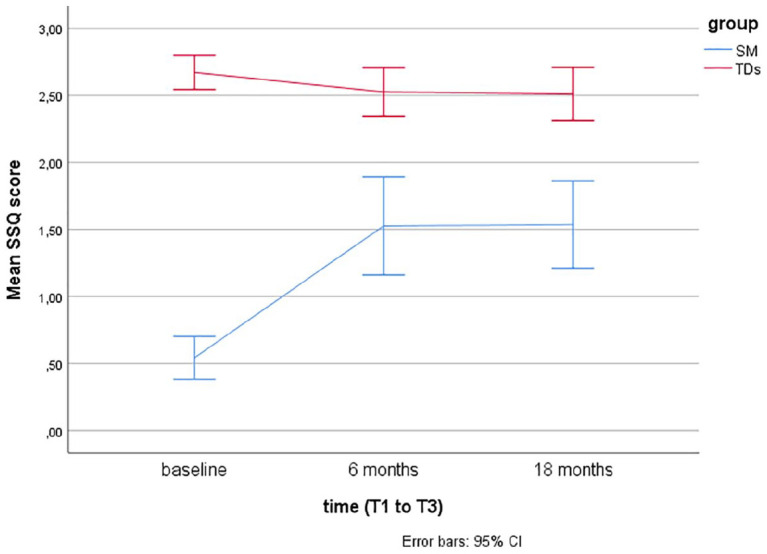
Mean School Speech Questionnaire (SSQ) scores over time (T1–T3) in children treated for Selective Mutism (SM) and typically developing children (TDs).

For children with SM, there was no significant increase in the SMQ Home and Public subscales or the SSQ from T2 (end of treatment) to T3 (at follow-up, after 18 months), but a significant increase was found in the SMQ School subscale (*p* = .02) and the SMQ total score (*p* = .008). The scores on the SM questionnaires over time (baseline, after 6 and 18 months) are presented for the two groups (TDs and SM) in Figures 1 to 4 (SMQ) and Figure 5 (SSQ). Raw scores can be found in the Supplementary File.

When including age group and gender as covariates in the analyses, we found no significant effect of either variable in TDs. In children treated for SM, there was no gender effect. However, age group had a significant effect, in that the younger children had better results at T2 on three of the measures (the SMQ School subscale, the SMQ Total score, and the SSQ, *p*’s < .001).

## Discussion

The SMQ and SSQ are the most commonly used standardized measures of speaking behavior in cross-sectional and longitudinal SM treatment studies. To better interpret findings of these studies, there was a need for more information on speaking behavior in TDs. The findings from this study support that the SMQ and SSQ taps into the core SM symptom, lack of speech, as TDs scored significantly higher than children with SM, as hypothesized, and in line with findings from two other studies with 16 (Bar-Haim et al., 2004) and 19 (Manassis et al., 2007) controls. The mean scores in TDs, as rated by parents and teachers, lie in the range from 2 = “often” to 3 = “always” compared with mean scores between 0 = “never” and 1 = “seldom” in children with SM (see Figures 1 to 5).

While the SMQ School and Home subscale scores in TDs are >2.5, close to 3.0, the mean SMQ Public subscale is close to 2.0 ( “often”). This relative difference, with somewhat less speech in public, is also found in children with SM, both in the initial SMQ psychometric study (Bergman et al., 2008), in treatment studies summarized here (Table 1), and in a study on comorbidity and family factors associated with SM (*n* = 29, aged 3–13 years, 58% girls; Buzzella et al., 2011).

Our mean score findings in the TDs are quite similar to previous findings from the small group (*n* = 18) of referred children diagnosed with anxiety disorders other than SM, where a total SMQ score >2.5 was reported (Bergman et al., 2008). The relatively comparable parent-rated scores between children with anxiety disorders in the Bergman study (Bergman et al., 2008) and the TDs in this study have also been reported on the more recently developed parent-rated FSSM (Gensthaler et al., 2018). In the FSSM study, it was demonstrated that children with social phobia had scores about twice as high as those for the TDs and children with other anxiety disorders, and about half as high as the scores for children with SM, thus adding important information on speaking behavior as reported by parents in this diagnostic group (Gensthaler et al., 2018).

As previously mentioned, two studies found no significant change in speaking behavior after 3 months in waitlist controls with SM, in contrast to a significant increase in children with SM who had been treated for 3 months (Bergman et al., 2013; Oerbeck et al., 2014).

This study found no significant change over time in TDs and significantly increased speech after treatment in the SM group using two independent raters. This suggests that treatment, and not time, improves speaking behavior. However, this needs to be corroborated in future studies. Furthermore, although the children with SM showed a significantly increased speech after treatment, they still lagged behind the TDs. Comparable end points (⩽2) on the SMQ/SSQ after treatment has also been reported in other studies of SM (Bergman et al., 2013; Catchpole et al., 2019; Cornacchio et al., 2019; Klein et al., 2017). At present, we do not know whether a more intensified treatment would increase speech in children with SM, or whether a somewhat reduced level of speech is to be expected in a group of children where behaviorally inhibited temperament is prominent (Gensthaler et al., 2016; Muris et al., 2016).

The lack of significant gender differences in both TDs and children with SM is in line with the previous SMQ psychometric studies (Bergman et al., 2008; Letamendi et al., 2008) supporting a similar use of the SM questionnaires in boys and girls. For children with SM, the fact that younger children had better results at T2 than the older children might be due to a possibly less entrenched mutism in younger subjects. However, as discussed in our long-term follow-up study (Oerbeck et al., 2018), we cannot rule out that our intervention is more suitable for younger children with SM. Maybe a form of active cognitive restructuring as a component of cognitive behavioral therapy (CBT) could be particularly important for older children, something that was not included in our study. Support for the latter is the beneficial effect also found in older children after a modular treatment of SM including a cognitive component (Lang et al., 2016).

### Strengths and limitations

The use of two different questionnaire raters (parents and teachers) and the inclusion of TDs as a matched control group to the children with SM are strengths of the study. Among the limitations is the lack of a structured diagnostic interview in the TDs, and future studies should include this information. However, a screening interview was performed, with no findings of psychopathology, and the children were non-referred and recruited by teachers as being normally functioning children. Another limitation when investigating changes over time is that the follow-up time ended after 18 months. However, this is a commonly used period for clinical treatment and follow-up studies in children with SM. Furthermore, there should ideally have been a third group with data over time, namely children with SM who did not undergo treatment. This was not possible for ethical reasons, as children referred to Norwegian CAMHS have a right to treatment after waiting maximum 3 months, and as previously noted, research has shown no change in speaking behavior among waitlist controls with SM over a 3-month period.

## Conclusion

We conclude that the SMQ and the SSQ discriminate well between children with SM and TDs. Our main finding of TDs being rated as speaking often or always (values 2 and 3 on the SMQ/SSQ), with no change over the investigated time period supports the questionnaires as good measures for severity of SM and effects of treatment in future studies.

## Supplemental Material

Supplementary_table__IDCCPP-19-0143_SM_questionnaires – Supplemental material for The Selective Mutism Questionnaire: Data from typically developing children and children with selective mutism Click here for additional data file. Supplemental material, Supplementary_table__IDCCPP-19-0143_SM_questionnaires for The Selective Mutism Questionnaire: Data from typically developing children and children with selective mutism by Beate Oerbeck, Kristin Romvig Overgaard, R. Lindsey Bergman, Are Hugo Pripp and Hanne Kristensen in Clinical Child Psychology and Psychiatry
