# Supplementary material for: The Selective Mutism Questionnaire: Data from typically developing children and children with selective mutism
Source: Clin Child Psychol Psychiatry. 2020 Apr 13;25(4):754–65. doi: 10.1177/1359104520914695 (PMC7528533; doi:10.1177/1359104520914695)
Supplement: Supplementary_table__IDCCPP-19-0143_SM_questionnaires – Supplemental material for The Selective Mutism Questionnaire: Data from typically developing children and children with selective mutism [file Supplementary_table__IDCCPP-19-0143_SM_questionnaires.pdf]

Supplementary table

Mean SMQ and SSQ results over time (T1-T3) in children with Selective Mutism (SM) and the typically developing children (TDs)

	**SM (n=32)**		**TDs (n=32)**	
**Parent rated**	**Mean**	**sd**	**Mean**	**sd**
SMQ_school_T1	0.53	0.43	2.66	0.29
SMQ_home_T1	1.73	0.66	2.86	0.21
SMQ_public_T1	0.34	0.44	2.23	0.37
SMQ_TOTAL_T1	0.87	0.36	2.58	0.23
SMQ_school_T2	1.45	0.89	2.56	0.34
SMQ_home_T2	2.25	0.56	2.84	0.24
SMQ_public_T2	0.91	0.69	2.19	0.42
SMQ_TOTAL_T2	1.58	0.61	2.55	0.26
SMQ_school_T3	1.67	0.84	2.62	0.31
SMQ_home_T3	2.45	0.46	2.80	0.26
SMQ_public_T3	1.14	0.83	2.16	0.43
SMQ_TOTAL_T3	1.79	0.62	2.55	0.27
**Teacher rated**	**Mean**	**sd**	**Mean**	**sd**
SSQ_T1	0.54	0.44	2.67	0.35
SSQ_T2	1.53	1.02	2.53	0.50
SSQ_T3	1.54	0.90	2.51	0.55

SMQ Selective Mutism Questionnaire; *SSQ* School Speech Questionnaire;
